# Impact of Different Low-Volume Concurrent Training Regimens on Cardiometabolic Health, Inflammation, and Fitness in Obese Metabolic Syndrome Patients

**DOI:** 10.3390/nu17030561

**Published:** 2025-01-31

**Authors:** Dejan Reljic, Hans Joachim Herrmann, Markus Friedrich Neurath, Yurdagül Zopf

**Affiliations:** 1 Department of Medicine 1, University Hospital Erlangen, Friedrich-Alexander University Erlangen-Nürnberg, 91054 Erlangen, Germany; hans.herrmann@uk-erlangen.de (H.J.H.); markus.neurath@uk-erlangen.de (M.F.N.); yurdaguel.zopf@uk-erlangen.de (Y.Z.); 2 Hector-Center for Nutrition, Exercise and Sports, Department of Medicine 1, University Hospital Erlangen, Friedrich-Alexander University Erlangen-Nürnberg, 91054 Erlangen, Germany; 3 Deutsches Zentrum Immuntherapie (DZI), University Hospital Erlangen, Friedrich-Alexander University Erlangen-Nürnberg, 91054 Erlangen, Germany

**Keywords:** low-volume concurrent training, low-volume HIIT, single-set resistance training, WB-EMS, interference effect, obesity

## Abstract

Background/Objectives: Evidence supports the benefits of concurrent training (CT), which combines endurance and resistance exercises, for enhancing health and physical fitness. Recently, low-volume, time-efficient exercise approaches such as low-volume high-intensity interval training (LOW-HIIT), whole-body electromyostimulation (WB-EMS), and single-set resistance training (1-RT) have gained popularity for their feasibility and efficacy in improving various health outcomes. This study investigated the effects of low-volume CT, focusing on (1) whether exercise order affects cardiometabolic health, inflammation, and fitness adaptations and (2) which combination, LOW-HIIT plus WB-EMS or LOW-HIIT plus 1-RT, yields better results. Methods: Ninety-three obese metabolic syndrome (MetS) patients undergoing caloric restriction were randomly assigned to four groups performing the different low-volume CT protocols over 12 weeks. Outcomes included cardiometabolic, inflammatory, and fitness parameters. Results: In both combinations, no significant differences were found regarding exercise order. However, the pooled LOW-HIIT and 1-RT group achieved superior improvements in blood pressure, blood lipids, inflammation markers (CRP, hsCRP), the MetS severity score, and overall fitness compared to the LOW-HIIT and WB-EMS combination. Compared to previous studies using these modalities individually, LOW-HIIT plus 1-RT appeared to further reduce inflammation, whereas LOW-HIIT combined with WB-EMS was less effective for cardiometabolic health, potentially due to interference effects between modalities. Conclusions: While LOW-HIIT plus WB-EMS appears to be a viable option for individuals unable to perform traditional resistance training, the findings suggest prioritizing LOW-HIIT plus 1-RT to maximize health outcomes. These findings highlight the importance of tailored exercise prescriptions and the need for further research into optimizing CT protocols for diverse populations.

## 1. Introduction

The global prevalence of obesity and metabolic syndrome (MetS) has risen dramatically in recent decades, posing a major public health challenge [1]. MetS is a cluster of cardiometabolic disorders that increase the risk of type 2 diabetes [2], cardiovascular disease [3], some types of cancer [4], and mortality [5]. A hallmark of MetS is low-grade chronic inflammation, marked by elevated levels of inflammatory mediators such as *C*-reactive protein (CRP), which contribute to disease progression and adverse outcomes [6]. Caloric restriction through dietary modifications [7,8] and physical exercise [9,10] are foundational components in the treatment of obesity and MetS. However, the optimal exercise regimens to address cardiometabolic outcomes and inflammation in obese individuals remain an area of ongoing investigation. Evidence suggests that exercise interventions combining endurance (ET) and resistance training (RT), often referred to as concurrent training (CT), may provide superior benefits compared to single-modality approaches [11,12,13]. However, traditional CT programs typically involve moderate- to high-volume exercise regimens and may thus not be feasible or sustainable for individuals with obesity or MetS [14]. Given that “lack of time” is among the most commonly cited barriers for exercise participation in general [15] and obese [16] populations, there has been growing interest in the development and application of more time-efficient, low-volume exercise protocols in recent years. As such, low-volume high-intensity interval training (LOW-HIIT) [17], whole-body electromyostimulation exercise (WB-EMS) [18], and single-set resistance training (1-RT) [19] have emerged as interesting options for health promotion and clinical exercise programs.

Although research from our laboratory [20,21,22,23] and other groups [24,25,26] has demonstrated that these low-volume exercise modalities can provide several health benefits, little is known about their combined effects in CT regimens. In this respect, it has been reported, for example, that CT can result in potential “interference effects”, where the adaptations to RT may be blunted by the concomitant execution of ET and vice versa [27,28,29]. This phenomenon has been linked to antagonistic adaptations between ET and RT [28] and appears to occur more likely during higher volumes of CT [29,30]. Furthermore, the specific modality [28,29,30] and sequence of exercise (when ET and RT are consecutively performed within the same exercise session) [31,32] have been found to be other contributing factors for the occurrence of interference effects. It is important to note, however, that the majority of previous CT studies used higher-volume exercise programs (e.g., prolonged ET combined with multiple-set RT) and primarily focused on physical fitness outcomes, leaving gaps in our understanding of how low-volume CT impacts cardiometabolic health and inflammation in clinical populations.

Therefore, this study aimed to address two major research questions in a cohort of obese MetS patients: (1) to examine whether the order of LOW-HIIT and WB-EMS/1-RT affects the responses to low-volume CT, and (2) to compare the impact of the two different low-volume CT combinations (i.e., LOW-HIIT and WB-EMS vs. LOW-HIIT and 1-RT) on cardiometabolic, inflammation, and physical fitness outcomes. We hypothesized that training order would influence outcomes, with LOW-HIIT followed by WB-EMS or 1-RT yielding greater improvements in cardiorespiratory fitness, and the reverse order favoring muscle strength gains. Moreover, we expected that LOW-HIIT combined with 1-RT would produce superior cardiometabolic health and fitness benefits compared to LOW-HIIT combined with WB-EMS, based on prior findings from our laboratory [20,22,23].

## 2. Materials and Methods

### 2.1. Study Design

Participants were randomly assigned to one of four groups: (A) LOW-HIIT followed by WB-EMS (LOW-HIIT+WB-EMS), (B) WB-EMS followed by LOW-HIIT (WB-EMS+LOW-HIIT), (C) LOW-HIIT followed by 1-RT (LOW-HIIT+1-RT), and (D) 1-RT followed by LOW-HIIT (1-RT+LOW-HIIT). All patients received standard-care dietary counseling for caloric restriction and participated in twice-weekly training sessions for 12 weeks. The primary outcome measure was the metabolic syndrome severity z-score (MetS z-score), which was calculated from distinctive cardiometabolic risk markers (see Section 2.3.4). Secondary outcomes of interest were markers of chronic inflammation (CRP and high-sensitivity CRP), individual cardiometabolic risk markers, physical fitness outcomes including maximum oxygen uptake (VO_2max_) and maximum strength values of key muscle groups, the overall fitness score (Fit-score, derived from VO_2max_ and the average maximum strength value across the five main muscle groups, as specified in Section 2.3.6), and body composition parameters. Outcome assessments were conducted at two time points: one week prior to the start of the intervention (baseline, T-1) and during the first week after the completion of the 12-week CT program (week 13, T-2).

Patients were allocated to the four training groups through a stratified randomization process based on their baseline VO_2max_ (<20, 20–25, >25 mL/kg/min), age (<45 years or ≥45 years), gender (male or female), and body mass index (BMI <35 or ≥35 kg/m^2^). Group allocation was managed using MinimPy software (GNU General Public License version 3.0) [33] to ensure a proper balance across the strata. An independent researcher, uninvolved in the data collection process, conducted the randomization. All participants provided written informed consent after being fully informed about the study objectives, in accordance with the Declaration of Helsinki. The study received ethical approval from the Medical Faculty Ethics Committee of Friedrich-Alexander University Erlangen-Nürnberg (approval number: 132_18B, approval date: 17 April 2018) and was registered with ClinicalTrials.gov (ID: NCT03710447).

The data analysis for this study followed a two-step approach: (1) In the first step, we analyzed whether the order of execution of LOW-HIIT and WB-EMS/1-RT influenced the patients’ responses to the exercise program. This analysis involved comparing changes in primary and secondary outcomes between LOW-HIIT+WB-EMS and WB-EMS+LOW-HIIT and between LOW-HIIT+1-RT and 1-RT+LOW-HIIT, respectively. (2) The second step involved comparing the overall effects of the two different low-volume CT combinations (LOW-HIIT and WB-EMS vs. LOW-HIIT and 1-RT). This step aimed to evaluate the impact of the specific low-volume exercise combination on improvements in cardiometabolic health, inflammation, and physical fitness outcomes.

### 2.2. Study Patients

The inclusion criteria specified participants aged 18 years or older, with a BMI ≥ 30 kg/m^2^, a clinical diagnosis of MetS according to established criteria [34], and a self-reported sedentary lifestyle as defined elsewhere [35]. Exclusion criteria were a clinical diagnosis of heart disease, cancer, severe orthopedic conditions, other significant health issues that could hinder safe participation in exercise, and pregnancy. Each patient’s eligibility was reviewed during an initial screening session with the principal investigator before enrollment in the study. Patients committed to not making significant lifestyle changes during the intervention to reduce potential confounding factors. Patients were recruited through advertisements posted in local newspapers and on social media platforms and flyers placed in surrounding medical practices. Individuals expressing interest were invited to contact the study team via email or phone to determine their eligibility.

Building on previously published data from our group [21], which demonstrated an effect size of *d* = 0.53 for the impact of LOW-HIIT on the primary outcome (MetS z-score) in a cohort of obese patients with MetS, we conducted an a priori sample size calculation. This calculation indicated that a sample size of *n* = 16 participants per group would be required to achieve a statistical power of 0.95 for a repeated measures ANOVA with a significance level of 5% (G*Power, version 3.1.9.2). Given the potential for even greater effects on the MetS z-score by combining LOW-HIIT with WB-EMS or 1-RT, respectively, we anticipated a higher overall effect on the primary outcome compared to LOW-HIIT alone. However, considering that obesity intervention studies often report attrition rates as high as 80%, we aimed to recruit a minimum of *n* = 25 participants per group to adequately account for potential dropouts.

### 2.3. Testing Procedures

Baseline assessments (T-1) were conducted one week prior to the start of the CT program, and post-intervention assessments (T-2) were carried out within the first week following the completion of the 12-week exercise period. To ensure adequate recovery, a minimum of three days was scheduled between the final exercise session and post-testing. Both T-1 and T-2 assessments were carried out at the same time of day to minimize potential circadian influences. For female participants, testing was performed within the same phase of their menstrual cycle to control for hormonal fluctuations. Patients were instructed to arrive after an overnight fast, abstain from alcohol, and avoid any vigorous physical activity for at least 24 h before testing. All measurements were conducted under standardized conditions (ambient temperature of 22–24 °C and humidity between 30–50%). At T-1, outcome measurements were preceded by a comprehensive medical clearance, including a review of medical history, a 12-lead resting electrocardiogram, and standard blood and urine analyses to ensure that the patients were safe to engage in the exercise program. All assessments and examinations were performed by investigators blinded to group allocation, ensuring unbiased data collection.

#### 2.3.1. Assessment of Blood Pressure

Upon arrival at our Research Center, patients were instructed to empty their bladder prior to the blood pressure assessment. Thereafter, they rested in a seated position for five minutes before the readings were taken. Blood pressure was measured using a validated automatic upper-arm device (M5 Professional, Omron, Mannheim, Germany) [36]. Two successive measurements were recorded on both arms at 60-s intervals. For later analysis, the average of the measurements from the arm with the higher blood pressure was used, following standard recommendations [37].

#### 2.3.2. Blood Sample Collection and Analysis

Blood samples were drawn from the patients’ antecubital vein using disposable cannulas (S-Monovette, Sarstedt, Nürmbrecht, Germany) through standard venipuncture procedures. The laboratory analyses involved measurements of serum concentrations of the inflammation markers CRP and high-sensitivity CRP (hsCRP) as well as of fasting glucose, triglycerides, total cholesterol, low-density lipoprotein (LDL) cholesterol, high-density lipoprotein (HDL) cholesterol, serum glycosylated hemoglobin A_1c_ (HbA_1c_), and insulin. All blood analyses were conducted at the Central Laboratories of the University Hospital Erlangen, adhering to protocols previously described in detail [22]. Additionally, the homeostasis model assessment of insulin resistance (HOMA-IR) index was calculated as a measure of insulin resistance according to the approach established by Matthews et al. [38].

#### 2.3.3. Assessment of Body Composition

Before the assessments, patients were instructed to sit again for five minutes to standardize measurements. Body composition outcomes, including body weight, body fat mass (FM), percentage of body fat (FM%), fat-free mass (FFM), and total body water (TBW), were then evaluated using a validated multi-frequency segmental bioelectrical impedance analysis device (seca mBCA 515, Seca, Hamburg, Germany) [39]. Additionally, waist circumference (WC) was measured with participants standing upright, using a flexible measuring tape positioned midway between the lower edge of the last palpable rib and the top of the iliac crest, along the mid-axillary line. The measurement was recorded to the nearest millimeter, following standard guidelines [40].

#### 2.3.4. Calculation of the Metabolic Syndrome z-Score

The MetS z-score is a continuous measure that quantifies the MetS severity, offering a more nuanced assessment of cardiometabolic risk. Unlike categorical definitions of MetS, which rely on threshold criteria for individual risk factors, such as blood pressure or blood lipid concentrations, the z-score approach allows for the detection of subtle, clinically significant changes that may not cross predefined cut-off points. This makes the z-score a more sensitive tool for evaluating overall cardiometabolic risk, as supported by previous research [41]. As described in more detail elsewhere [42], the MetS z-score was computed using sex-specific equations that consider the following key cardiometabolic variables: WC, mean arterial pressure (MAB), fasting serum glucose, triglycerides, and HDL.

#### 2.3.5. Assessment of Cardiorespiratory Fitness Outcomes

Cardiopulmonary exercise testing (CPET) was performed using an electronically controlled cycle ergometer (Corival CPET, Lode, Groningen, The Netherlands) to evaluate key cardiorespiratory parameters, including VO_2max_, maximum power output (W_max_), and maximum heart rate (HR_max_). Additionally, power output at the ventilatory threshold (W_VT_) was determined using the V-Slope method by analyzing the ratio of carbon dioxide output (VCO_2_) to oxygen consumption (VO_2_) [43]. After a brief 1-min familiarization period, patients began cycling at an initial workload of 50 W. Using a standardized ramp protocol, the power output was increased incrementally—12.5 W/min for females and 15 W/min for males—until the patients reached voluntary exhaustion. Exhaustion was typically achieved within a time frame of 8 to 12 min, which is in line with established CPET recommendations [35]. Heart rate was continuously monitored using a 12-lead electrocardiogram (custo cardio 110, custo med, Ottobrunn, Germany), while VO_2_ and VCO_2_ were measured on a breath-by-breath basis with a metabolic cart (Metalyzer 3B-R3, Cortex Biophysik, Leipzig, Germany). To confirm that patients had reached maximal exertion, at least two of the following criteria were required [44]: a plateau in VO_2_, respiratory exchange ratio (RER) of at least 1.10 or higher, HR_max_ exceeding 90% of the age-predicted maximum (calculated as 220 minus age), or a perceived exertion rating of at least 19 or higher on the Borg scale [45]. The CPET data were subsequently used to define individualized heart rate zones for the LOW-HIIT.

#### 2.3.6. Assessment of One-Repetition Maximum Strength and Overall Fitness z-Score

Prior to the strength tests, patients familiarized themselves with the testing procedures and performed localized warm-up exercises targeting the specific muscle groups. A modified version of the one-repetition maximum (1-RM) test was used to assess the strength of the five major groups (i.e., legs, abdominals, chest, upper back, and lower back). Unlike the traditional 1-RM test, which determines the maximum weight lifted for a single repetition, the modified protocol used multiple repetitions to estimate 1-RM values. This approach is considered safer for individuals with limited training experience and is recommended for reducing injury risk [46]. The strength assessments were conducted on five different resistance machines, in the following standardized order: (1) chest press, (2) lat pulldown, (3) lower back extension, (4) abdominal crunch, and (5) leg press (TechnoGym, Neu-Isenburg, Germany). Each patient was supervised by a certified physiotherapist or sports therapist. On each machine, patients were required to lift the respective weight until they reached muscular failure, with a target of no more than six repetitions to ensure accurate 1-RM prediction, as suggested elsewhere [47]. If more than six repetitions were achieved, the weight was increased, and the test was repeated after a 3-min rest. Typically, the appropriate load was determined within three attempts. The estimated 1-RM was calculated using a previously published formula [48], allowing for strength predictions based on submaximal efforts. These results were used to set the individual weight loads for the RT exercises in the 1-RT groups. To account for training progress, the 1-RM test was repeated at the beginning of the fifth and ninth week of the intervention to adjust training loads accordingly. Additionally, an overall fitness score (Fit-score) was calculated at T-1 and T-2 based on the average key measures of cardiorespiratory and muscular fitness according to the following formula:

Fit-score = (VO_2max_ + average 1-RM from the five muscle groups)/2

### 2.4. Nutritional Guidance

At the start of the study, patients met individually with a registered dietitian for personalized nutritional counseling. Following international obesity treatment guidelines [8], they were advised to aim for a daily caloric deficit of 500 kcal, while ensuring adequate protein intake (≥1.0 g/kg/day) to help preserve skeletal muscle mass during caloric restriction [49]. To assist with meal planning and implementation of these guidelines, patients received handouts detailing suggested meal plans and a list of foods with their corresponding calorie values. Throughout the study, patients had ongoing access to the nutrition team for additional consultations if they needed clarification or support in following their dietary regimen. Dietary intake was tracked using 24-h dietary records (Freiburger Ernährungsprotokoll; Nutri-Science, Freiburg, Germany), completed over three consecutive days at both the beginning of the study and during the final week of the intervention. The average caloric intake and nutrient composition from these records were analyzed with PRODI 6 expert software (Nutri-Science, Freiburg, Germany) to assess adherence to the dietary goals.

### 2.5. Low-Volume Concurrent Training Program

Over a 12-week period, patients underwent two CT sessions per week, amounting to a total of 24 sessions. Flexibility in scheduling was provided by allowing patients to book their exercise sessions individually throughout the operational hours of the Training Center, with at least 24 h of rest between sessions for adequate recovery. Depending on the group assignment, the exercise combinations involved one of the following protocols: (A) LOW-HIIT followed by WB-EMS; (B) WB-EMS preceding LOW-HIIT; (C) LOW-HIIT followed by 1-RT; or (D) 1-RT preceding LOW-HIIT. All sessions were supervised by certified sports therapists or physiotherapists to ensure correct execution and safety.

The LOW-HIIT protocol was executed on cycle ergometers, following the approach established by Reljic et al. [50]. Briefly, the protocol commenced with a 2-min warm-up at a light cycling pace. Patients then performed five exercise intervals lasting 1 min each. In weeks 1–4, intervals were performed at a minimum of 80–85% of the patient’s HR_max_, progressing to 85–90% HR_max_ in weeks 5–8, and reaching 90–95% HR_max_ in weeks 9–12. Throughout the training, patients wore chest-strap heart rate monitors (acentas, Hörgertshausen, Germany) for real-time tracking. For each session, heart rate data were saved and later analyzed using dedicated software (HR monitoring team system, acentas, Hörgertshausen, Germany). To maintain their individual target heart rate zones, patients were directed to adjust the watt load and/or pedaling cadence during the intervals. Each interval was followed by a 1-min active recovery with low-intensity cycling, and the last interval concluded with a 3-min cool-down at a self-selected pace. In line with prior definitions of low-volume HIIT [17], the total duration per each LOW-HIIT session (including warm-up and cool-down) was 14 min.

The WB-EMS protocol involved the use of specific equipment from miha bodytec (Gersthofen, Germany), comprising a vest, hip belt, and cuffs for the arms and thighs, all fitted with in-built electrodes to deliver electrical muscle stimulation. The stimulation parameters included a frequency of 85 Hz and a pulse width of 350 μs, delivered in 6-s impulses followed by a 4-s rest period, as typically employed in most WB-EMS applications [25]. Eight major muscle groups were stimulated, including the arms, chest, upper back, latissimus, abdomen, lower back, buttocks, and thighs. During each stimulation phase, patients engaged in slow dynamic movements (e.g., trunk flexions, light squats, and arm exercises), performing two sets of ten repetitions each, to further activate the targeted muscles. The intensity of the electrical stimulation was adjusted throughout the training to elicit strong muscle contractions and ensure progressive overload. Each WB-EMS protocol lasted 20 min.

For the 1-RT protocol, five resistance exercises were performed targeting major muscle groups using specific strength machines (TechnoGym, Neu-Isenburg, Germany), including the chest press, lat pulldown, lower back machine, abdominal crunch, and leg press. A single set of each exercise was completed with a resting period of 2 min before proceeding to the next device. During the first four weeks, patients performed each exercise at 50–60% of their 1-RM, aiming for 15–20 repetitions to allow for an adaptation phase. In weeks 5–8, the load was increased to 60–70% of 1-RM, with a target of 10–15 repetitions per exercise, and during weeks 9–12, the intensity was further raised to 70–80% of 1-RM to achieve 8–12 repetitions. Overall, each session, including LOW-HIIT and either WB-EMS or 1-RT, lasted approximately 30–35 min, equating to about 60–70 min of exercise per week.

### 2.6. Statistical Analysis

All statistical analyses were conducted using SPSS version 24.0 (SPSS Inc., Chicago, IL, USA). Initially, the Shapiro–Wilk test was applied to test for normality in the data. The subsequent analyses followed a two-step approach to evaluate the different research questions. First, in order to assess whether the order of performing LOW-HIIT and WB-EMS or 1-RT influenced patients’ responses, two separate 2 × 2 repeated measures ANOVAs were used to examine the main effects of the exercise order in LOW-HIIT+WB-EMS vs. WB-EMS+LOW-HIIT and LOW-HIIT+1-RT vs. 1-RT+LOW-HIIT, respectively, as well as the respective time (pre- vs. post-intervention) and interaction effects. In the second step, the overall effects of the two different low-volume CT combinations (i.e., the pooled data of the two LOW-HIIT and WB-EMS groups vs. the two LOW-HIIT and 1-RT groups) were compared. A 2 × 2 repeated measures ANOVA was conducted to assess the main effects of group (LOW-HIIT and WB-EMS vs. LOW-HIIT and 1-RT) and time (pre- vs. post-intervention), as well as their interaction. In addition, ANCOVAs were performed to check for any influence of gender and age on changes in study outcomes.

In both steps, significant ANOVA results were followed by Holm–Sidak’s post hoc tests to examine the between-group differences, and paired *t*-tests were used to evaluate within-group differences. In both steps, Levene’s test was used to assess the homogeneity of variance prior to ANOVAs. When the data violated normality assumptions, logarithmic or square root transformations were applied for analyses. Subsequently, the transformed data were back-transformed to their original scale to facilitate interpretation and ensure that reported values were presented in their original units. If transformation failed to normalize the data, non-parametric tests, such as Friedman two-way analyses of variance, were used, followed by Dunn’s Bonferroni post hoc tests for comparisons between groups and Wilcoxon’s tests for comparisons within groups, respectively.

Effect sizes were calculated using partial eta-squared (*ηp*^2^) for ANOVA-based results, with thresholds for small (≤0.01), medium (≥0.06), and large (≥0.14) effects, and for the Friedman tests, Kendall’s W was computed to assess effect size, with thresholds of small (≤0.10), medium (≥0.30), and large (≥0.50) [51]. Statistical significance for all tests was set at *p* < 0.05. Data are reported as means ± standard deviation (SD), and changes between pre- and post-intervention are presented with 95% confidence intervals (95% CI).

## 3. Results

### 3.1. Study Flow, Training Data, and Adverse Events

In total, 196 individuals were screened for eligibility. Twelve patients were excluded for not meeting the inclusion criteria, and three withdrew due to loss of interest. After the baseline health examination, four patients were excluded due to detected medical issues precluding participation, and two withdrew for personal reasons. Thus, 177 participants were randomized to (A) LOW-HIIT+WB-EMS (*n* = 30), (B) WB-EMS+LOW-HIIT (*n* = 31), (C) LOW-HIIT+1-RT (*n* = 30), (D) 1-RT+LOW-HIIT (*n* = 30), or (E) other exercise groups (*n* = 56, not included in the analysis as they were not part of this specific study objective). The main characteristics of all included patients are shown in Table 1. Analysis of the data indicated no significant differences in main baseline characteristics among the four groups. Furthermore, no significant effects of gender on changes in study outcomes were detected (all *p*-values > 0.05). The data from female and male patients were therefore analyzed together.

**Figure 1:**
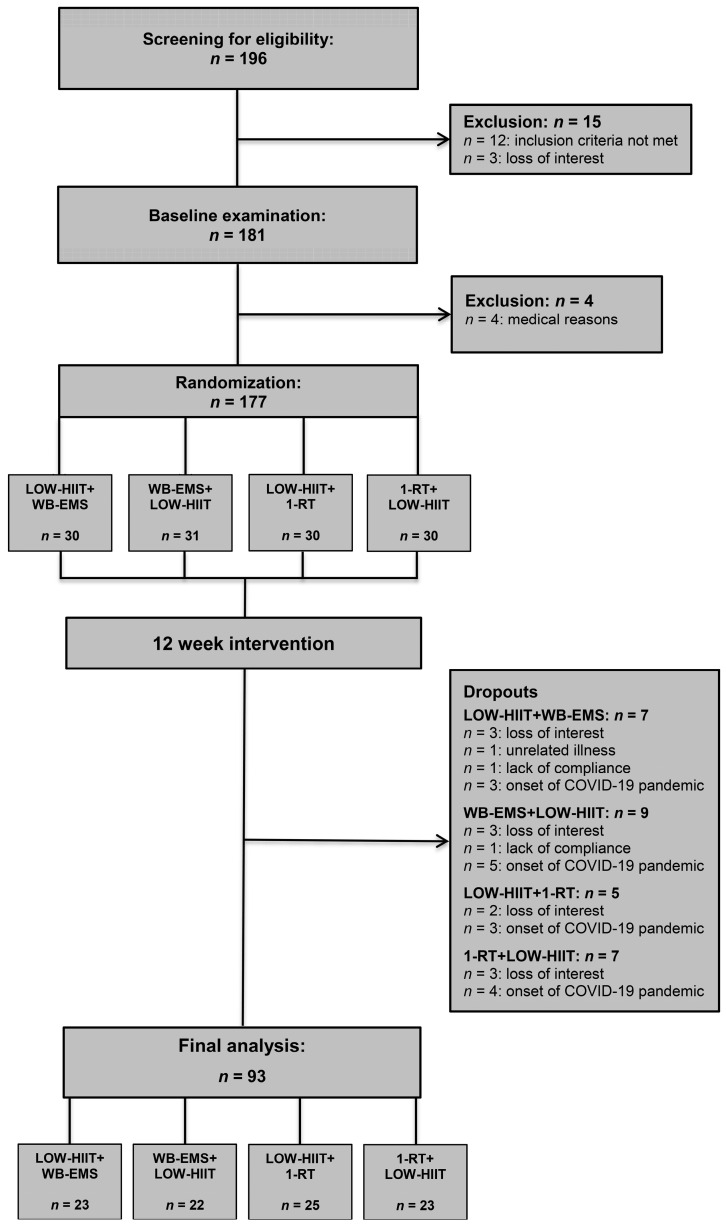
Study flow chart.

During the study, a total of 28 patients dropped out (LOW-HIIT+WB-EMS, *n* = 7; WB-EMS+LOW-HIIT, *n* = 9; LOW-HIIT+1-RT, *n* = 5; 1-RT+LOW-HIIT, *n* = 7). The specific reasons for dropout are illustrated in Figure 1. Thus, the study concluded with a total of 93 patients who had been analyzed. Baseline data on concomitant diseases and medication for the final study sample are summarized in Supplementary Table S1. Training adherence, measured by the percentage of scheduled sessions attended, was consistently high across all groups LOW-HIIT+WB-EMS (95 ± 8%), WB-EMS+LOW-HIIT (94 ± 8%), LOW-HIIT+1-RT (94 ± 9%), and 1-RT+LOW-HIIT (95 ± 8%).

The intended training intensity for LOW-HIIT was effectively achieved in all groups, as evidenced by the average peak heart rate recorded during the intervals, which corresponded to 96 ± 3%, 94 ± 5%, 94 ± 4%, and 95 ± 3% of the patients’ HR_max_ for LOW-HIIT+WB-EMS, WB-EMS+LOW-HIIT, LOW-HIIT+1-RT, and 1-RT+LOW-HIIT. Furthermore, the overall mean heart rate throughout the LOW-HIIT sessions—including warm-up, intervals, recovery phases, and cool-down—was consistent across groups, averaging 81 ± 5% (LOW-HIIT+WB-EMS), 80 ± 5% (WB-EMS+LOW-HIIT), 80 ± 5% (LOW-HIIT+1-RT), and 81 ± 4% (1-RT+LOW-HIIT) of HR_max_. Progression in resistance loads for 1-RT and electrical current for WB-EMS was systematically achieved among all patients over the intervention period, following the prescribed progression plan detailed in Section 2.5.

During the intervention period, only a few minor adverse events potentially associated with the exercise program were documented. These were all of an orthopedic nature and disappeared within a few days. The type and number of occurrences of adverse events for each group were as follows: LOW-HIIT+WB-EMS (mild back pain: *n* = 1, mild muscle cramps: *n* = 2, mild foot pain: *n* = 2), WB-EMS+LOW-HIIT (mild knee pain: *n* = 2, mild leg pain: *n* = 1), LOW-HIIT+1-RT (mild knee pain: *n* = 1), and 1-RT+LOW-HIIT (mild back pain: *n* = 1, mild knee pain: *n* = 2, mild shoulder pain: *n* = 1). The patients rated the training programs highly in terms of enjoyment, with average scores on a 7-point Likert scale (1 = “not enjoyable at all”; 7 = “extremely enjoyable”) of 5.9 ± 0.9 for LOW-HIIT+WB-EMS, 6.0 ± 0.7 for WB-EMS+LOW-HIIT, 5.9 ± 0.7 for LOW-HIIT+1-RT, and 6.1 ± 0.9 for 1-RT+LOW-HIIT. Importantly, 92% of patients across all groups (with no significant differences between groups) expressed a willingness to continue the exercise regimen beyond the study period.

### 3.2. Nutritional Intakes in All Groups

At both measurement points, no significant differences in diet were observed among the four groups. However, a significant effect of time was noted for energy intake (*p* < 0.001, *ή*^2^ = 0.63), fat (*p* < 0.001, *ή*^2^ = 0.24), and carbohydrate consumption (*p* < 0.001, *ή*^2^ = 0.34). Across all groups, there was a significant reduction in total daily energy intake, primarily driven by lower fat and carbohydrate consumption. Table 2 provides a detailed overview of dietary intake for all groups, recorded during the week before the study began and during the final week of training.

The analysis of the combined data from the LOW-HIIT+WB-EMS and WB-EMS+LOW-HIIT groups versus the LOW-HIIT+1-RT and 1-RT+LOW-groups revealed no significant between-group differences in energy and macronutrient intake. Therefore, the pooled data for the combined groups are not shown separately.

### 3.3. Study Part 1: Impact of Exercise Order in LOW-HIIT and WB-EMS

#### 3.3.1. Anthropometric Variables in LOW-HIIT and WB-EMS Groups

No significant interaction or group effects were identified for any of the anthropometric variables. However, significant main effects of time were observed for body weight (*p* = 0.020, *ή*^2^ = 0.12), BMI (*p* = 0.021, *ή*^2^ = 0.12), FM (*p* = 0.045, *ή*^2^ = 0.09), and WC (*p* = 0.020, *ή*^2^ = 0.12). Post hoc analyses revealed significant reductions in body weight and BMI in both groups. Reductions in FM and WC achieved statistical significance only in the WB-EMS+LOW-HIIT group. A summary of group-specific anthropometric data before and after the intervention is presented in Table 3.

#### 3.3.2. Cardiometabolic and Inflammation Variables in LOW-HIIT and WB-EMS Groups

No significant group effects were observed for any of the cardiometabolic or inflammation-related variables. However, significant main effects of time were identified for resting heart rate (*p* = 0.035, *ή*^2^ = 0.10) and glucose levels (*p* = 0.011, *ή*^2^ = 0.14). Additionally, a significant interaction effect was detected for hsCRP levels. A trend toward a significant interaction effect was noted for CRP levels (*p* = 0.052). Post hoc analyses indicated a significant reduction in resting heart rate and a significant increase in glucose levels in the LOW-HIIT+WB-EMS group between T-1 and T-2. Furthermore, post hoc analyses revealed a significant reduction in hsCRP concentrations in the LOW-HIIT+WB-EMS group, but no significant differences in pre-/post-intervention changes were found between the groups. Comprehensive pre- and post-intervention data for all cardiometabolic and inflammation markers are detailed in Table 3.

#### 3.3.3. Physical Fitness Variables in LOW-HIIT and WB-EMS Groups

There were no significant interaction or group effects for any physical fitness variable. Regarding CPET-derived variables, significant main effects of time were found for relative VO_2max_ (*p* < 0.001, *ή*^2^ = 0.17), absolute VO_2max_ (*p* < 0.001, *ή*^2^ = 0.17), W_max_ (*p* < 0.001, *ή*^2^ = 0.24), and W_VT_ (*p* < 0.001, *ή*^2^ = 0.16). In strength tests, there were significant time effects for 1-RM of the chest (*p* < 0.001, *ή*^2^ = 0.42), upper back (*p* < 0.001, *ή*^2^ = 0.52), abdominals (*p* < 0.001, *ή*^2^ = 0.50), lower back (*p* = 0.001, *ή*^2^ = 0.26), and legs (*p* < 0.001, *ή*^2^ = 0.28). Moreover, a significant main effect of time was found for the Fit-score (*p* < 0.001, *ή*^2^ = 0.49).

For both, LOW-HIIT+WB-EMS and WB-EMS+LOW-HIIT, post hoc tests showed significant improvements in relative and absolute VO_2max_, W_max_, and W_VT_. Additionally, 1-RM values of the chest, upper back, abdominals, lower back, and legs, as well as the Fit-score, increased significantly in both groups. All group-specific physical fitness variables determined at T-1 and T-2 are presented in Table 3.

### 3.4. Study Part 1: Impact of Exercise Order in LOW-HIIT and 1-RT

#### 3.4.1. Anthropometric Variables in LOW-HIIT and 1-RT Groups

No significant interaction or group effects were detected for any anthropometric variables. However, significant main effects of time were identified for body weight (*p* < 0.001, *ή*^2^ = 0.22), BMI (*p* < 0.001, *ή*^2^ = 0.25), FM (*p* = 0.003, *ή*^2^ = 0.18), FM% (*p* = 0.014, *ή*^2^ = 0.13), and WC (*p* < 0.001, *ή*^2^ = 0.42). In both the LOW-HIIT+1-RT and 1-RT+LOW-HIIT groups, post hoc analyses demonstrated significant reductions in body weight, BMI, FM, and WC. Furthermore, a significant reduction in FM% was observed in the 1-RT+LOW-HIIT group. Detailed pre- and post-intervention anthropometric data for each group are summarized in Table 4.

#### 3.4.2. Cardiometabolic and Inflammation Variables in LOW-HIIT and 1-RT Groups

No significant interaction or group effects were observed for cardiometabolic or inflammation-related variables. However, significant main effects of time were identified for resting heart rate (*p* = 0.002, *ή*^2^ = 0.18), systolic blood pressure (*p* < 0.001, *ή*^2^ = 0.38), diastolic blood pressure (*p* < 0.001, *ή*^2^ = 0.52), MAB (*p* < 0.001, *ή*^2^ = 0.52), HbA_1c_ (*p* = 0.017, *ή*^2^ = 0.14), CRP (*p* < 0.001, *ή*^2^ = 0.22), hsCRP (*p* < 0.001, *ή*^2^ = 0.22), triglycerides (*p* = 0.024, *ή*^2^ = 0.11), HDL (*p* = 0.021, *ή*^2^ = 0.11), insulin (*p* = 0.033, *ή*^2^ = 0.10), HOMA-IR (*p* = 0.044, *ή*^2^ = 0.09), and the MetS z-score (*p* < 0.001, *ή*^2^ = 0.56).

Post hoc analyses showed significant improvements in both the LOW-HIIT+1-RT and 1-RT+LOW-HIIT groups. These included reductions in resting heart rate, systolic and diastolic blood pressure, MAB, CRP, hsCRP, and the MetS z-score. Additionally, in the 1-RT+LOW-HIIT group, post hoc tests revealed significant reductions in HbA_1c_, triglycerides, insulin, and HOMA-IR, alongside an increase in HDL. Comprehensive pre- and post-intervention data for all cardiometabolic and inflammation markers are presented in Table 4.

#### 3.4.3. Physical Fitness Variables in LOW-HIIT and 1-RT Groups

No significant interaction or group effects were observed for any physical fitness variables. However, significant main effects of time were identified for all CPET-derived variables: relative VO_2max_ (*p* < 0.001, *ή*^2^ = 0.45), absolute VO_2max_ (*p* < 0.001, *ή*^2^ = 0.28), W_max_ (*p* < 0.001, *ή*^2^ = 0.80), and W_VT_ (*p* < 0.001, *ή*^2^ = 0.66). In strength assessments, significant time effects were noted for the 1-RM of the chest (*p* < 0.001, *ή*^2^ = 0.58), upper back (*p* < 0.001, *ή*^2^ = 0.69), abdominals (*p* < 0.001, *ή*^2^ = 0.66), lower back (*p* = 0.001, *ή*^2^ = 0.53), and legs (*p* < 0.001, *ή*^2^ = 0.54). Additionally, a significant main effect of time was observed for the Fit-score (*p* < 0.001, *ή*^2^ = 0.49).

In both the LOW-HIIT+1-RT and 1-RT+LOW-HIIT groups, post hoc tests revealed significant improvements in relative and absolute VO_2max_, W_max_, and W_VT_. Strength measures also showed significant increases in 1-RM values of the chest, upper back, abdominals, lower back, and legs in both groups. Additionally, the Fit-score improved in both groups. All group-specific physical fitness data measured at T-1 and T-2 are summarized in Table 4.

### 3.5. Study Part 2: Comparison of LOW-HIIT and WB-EMS Versus LOW-HIIT and 1-RT

As no significant group effects were found between LOW-HIIT+WB-EMS and WB-EMS+LOW-HIIT and between LOW-HIIT+1-RT and 1-RT+LOW-HIIT, respectively, the data from the four subgroups were pooled into two groups for the second part of the study and compared with each other: the two LOW-HIIT and WB-EMS groups versus the two LOW-HIIT and 1-RT groups.

#### 3.5.1. Comparison of Anthropometric Variables in the Pooled Groups

Significant main effects of time were observed for several anthropometric measures, including body weight (*p* < 0.001, *ή*^2^ = 0.18), BMI (*p* < 0.001, *ή*^2^ = 0.19), FM (*p* < 0.001, *ή*^2^ = 0.13), FM% (*p* = 0.013, *ή*^2^ = 0.07), and WC (*p* < 0.001, *ή*^2^ = 0.28). In addition, there was also a significant interaction effect identified for WC (*p* = 0.005, *ή*^2^ = 0.08). Post hoc analyses revealed significant reductions in body weight, BMI, FM, and WC across the pooled LOW-HIIT and WB-EMS and LOW-HIIT and 1-RT groups. A significant reduction in FM% was only observed in the pooled LOW-HIIT and 1-RT group. However, no significant differences in the pre–post changes were detected when comparing the two pooled groups.

#### 3.5.2. Comparison of Cardiometabolic and Inflammation Variables in the Pooled Groups

Significant main effects of time were observed for several key cardiometabolic and inflammation parameters, including resting heart rate (*p* < 0.001, *ή*^2^ = 0.14), systolic blood pressure (*p* < 0.001, *ή*^2^ = 0.17), diastolic blood pressure (*p* < 0.001, *ή*^2^ = 0.21), MAB (*p* < 0.001, *ή*^2^ = 0.24), HbA_1c_ (*p* = 0.011, *ή*^2^ = 0.07), CRP (*p* < 0.001, *ή*^2^ = 0.16), hsCRP (*p* < 0.001, *ή*^2^ = 0.16), and the MetS z-score (*p* < 0.001, *ή*^2^ = 0.22). Notably, interaction effects were also identified for multiple variables, including systolic blood pressure (*p* = 0.025, *ή*^2^ = 0.05), diastolic blood pressure (*p* = 0.011, *ή*^2^ = 0.07), MAB (*p* = 0.009, *ή*^2^ = 0.07), glucose (*p* = 0.027, *ή*^2^ = 0.05), CRP (*p* = 0.033, *ή*^2^ = 0.05), hsCRP (*p* = 0.026, *ή*^2^ = 0.06), triglycerides (*p* = 0.046, *ή*^2^ = 0.04), HDL (*p* = 0.015, *ή*^2^ = 0.06), and the MetS z-score (*p* < 0.001, *ή*^2^ = 0.33).

**Figure 2:**
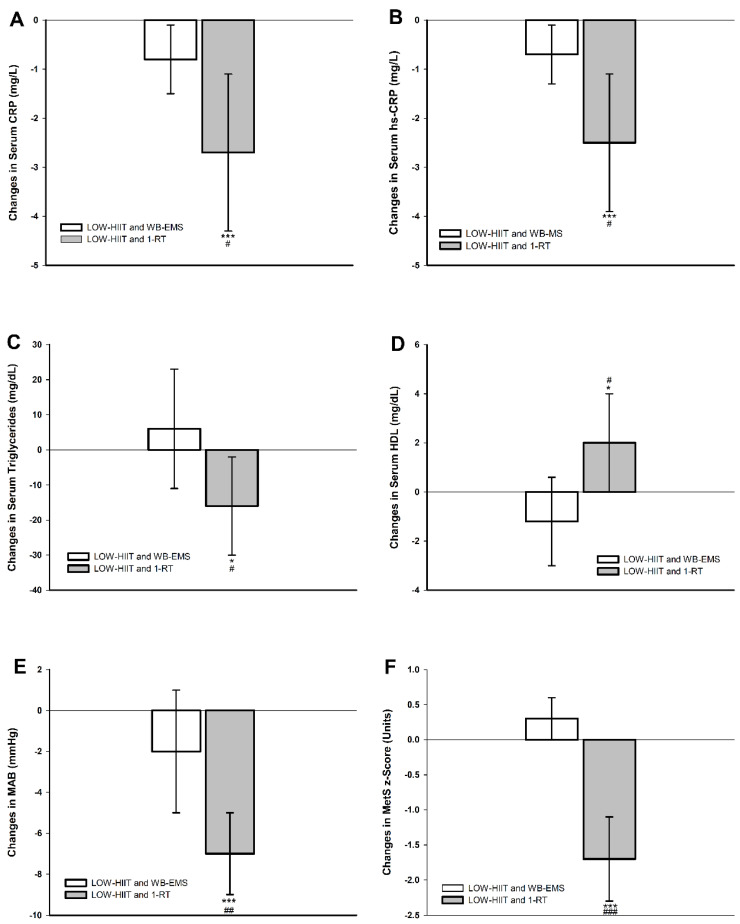
Changes in (**A**) serum *C*-reactive protein (CRP), (**B**) serum high-sensitivity *C*-reactive protein (hsCRP), (**C**) serum triglycerides, (**D**) serum high-density lipoprotein cholesterol (HDL), (**E**) mean arterial blood pressure (MAB), (**F**) metabolic syndrome severity z-score (MetS z-score). * (*p* < 0.01), *** (*p* < 0.001): significant change between T-1 and T-2. ^#^ (*p <* 0.05), ^##^ (*p <* 0.01), ^###^ (*p <* 0.001): significant difference between LOW-HIIT and 1-RT and LOW-HIIT and WB-EMS.

Post hoc analyses revealed significant reductions in resting heart rate for both pooled groups. Improvements in other parameters, however, were only observed in the pooled LOW-HIIT and 1-RT group, including significant changes in systolic and diastolic blood pressure, MAB, HbA_1c_, CRP, hsCRP, triglycerides, HDL, and the MetS z-score. Further, post hoc group comparisons revealed that the pooled LOW-HIIT and 1-RT group achieved significantly greater reductions in systolic blood pressure (−6 mmHg, *p* = 0.025), diastolic blood pressure (−5 mmHg, *p* = 0.011), MAB (−5 mmHg, *p* = 0.009), CRP (−1.9 g/L, *p* = 0.031), hsCRP (−1.8 g/L, *p* = 0.024), triglycerides (−22 mg/dL, *p* = 0.046), and the MetS z-score (−1.9 units, *p* < 0.001), and significantly greater increases in HDL (+3 mg/dL, *p* = 0.015), respectively, compared to the pooled LOW-HIIT and WB-EMS group. These group differences are visually presented in Figure 2.

#### 3.5.3. Comparison of Physical Fitness Variables in the Pooled Groups

Significant main effects of time were identified across all cardiorespiratory fitness-related variables, including relative VO_2max_ (*p* < 0.001, *ή*^2^ = 0.37), absolute VO_2max_ (*p* < 0.001, *ή*^2^ = 0.27), W_max_ (*p* < 0.001, *ή*^2^ = 0.73), and W_VT_ (*p* < 0.001, *ή*^2^ = 0.57). Strength assessments also showed significant time effects for 1-RM in several muscle groups: chest (*p* < 0.001, *ή*^2^ = 0.49), upper back (*p* < 0.001, *ή*^2^ = 0.61), abdominals (*p* < 0.001, *ή*^2^ = 0.59), lower back (*p* = 0.001, *ή*^2^ = 0.39), and legs (*p* < 0.001, *ή*^2^ = 0.41). Furthermore, a significant main effect of time was detected for the Fit-score (*p* < 0.001, *ή*^2^ = 0.63). Moreover, group-by-time interaction effects were significant for W_max_ (*p* = 0.012, *ή*^2^ = 0.07), W_VT_ (*p* = 0.019, *ή*^2^ = 0.06), abdominal strength (*p* = 0.002, *ή*^2^ = 0.11), lower back strength (*p* = 0.043, *ή*^2^ = 0.05), and the Fit-score (*p* = 0.041, *ή*^2^ = 0.05).

**Figure 3:**
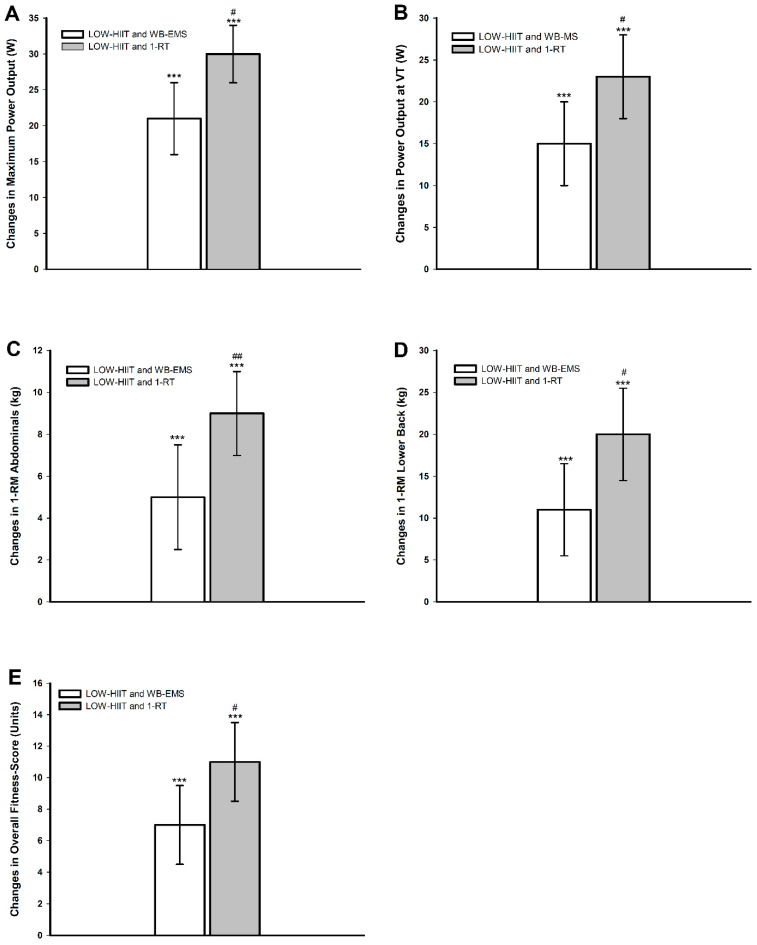
Changes in (**A**) maximum power output (W_max_), (**B**) power output at ventilatory threshold (W_VT_), (**C**) abdominals 1-RM, (**D**) lower back 1-RM, (**E**) overall physical fitness score (Fit-score). *** (*p* < 0.001): significant change between T-1 and T-2. ^#^ (*p* < 0.05), ^##^ (*p* < 0.01): significant difference between LOW-HIIT and 1-RT versus LOW-HIIT and WB-EMS.

Post hoc analyses confirmed significant improvements in relative and absolute VO_2max_, W_max_, and W_VT_ in both pooled groups. Strength improvements followed a similar pattern. In both pooled groups, significant 1-RM increases were observed for all muscle groups. Moreover, the Fit-score also improved significantly in both pooled groups. Importantly, the pooled LOW-HIIT and 1-RT group exhibited significantly larger pre-/post-intervention gains in specific parameters compared to the pooled LOW-HIIT and WB-EMS group. These included W_max_ (+8 W, *p* = 0.012), W_VT_ (+8 W, *p* = 0.019), abdominal strength (+4 kg, *p* = 0.002), lower back strength (+8 kg, *p* = 0.043), and the Fit-score (+3 units, *p* = 0.041). Figure 3 illustrates the significant differences in group-specific changes between T-1 and T-2.

### 3.6. Comparison of Changes in Inflammation and Cardiometabolic Variables in the Low-Volume CT Programs Versus Single-Modality Low-Volume Training Programs from Previous Research

**Figure 4:**
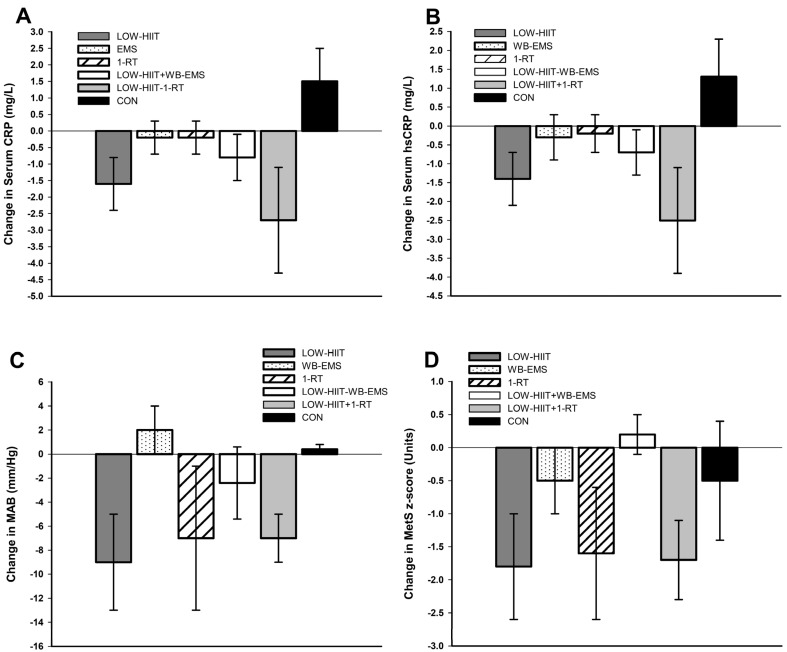
Changes in (**A**) serum *C*-reactive protein (CRP), (**B**) serum high-sensitivity *C*-reactive protein (hsCRP), (**C**) mean arterial blood pressure (MAB), and (**D**) metabolic syndrome severity z-score (MetS z-score) observed in the present trial in the pooled LOW-HIIT and WB-EMS and LOW-HIIT and 1-RT groups, respectively, compared to a previous study investigating the changes following LOW-HIIT, WB-EMS, 1-RT alone, or a non-active control condition (CON) [20]. Note: No statistical comparisons were performed as the results originate from different studies.

Figure 4 illustrates changes in inflammation, MAB, and MetS severity observed in the present study compared to findings from a previous study in which similar MetS populations underwent LOW-HIIT, WB-EMS, or 1-RT alone under identical conditions [20]. As no direct statistical comparisons were conducted between these groups (due to their origins from different studies), the data in Figure 4 are provided solely for illustrative purposes. These findings are discussed further in the Discussion section.

## 4. Discussion

To the best of our knowledge, this study is the first to evaluate the impact of different low-volume CT regimens on cardiometabolic, inflammation, and physical fitness outcomes in obese patients with MetS. Two major findings emerged: (1) Contrary to our hypothesis, training order (LOW-HIIT followed by WB-EMS/1-RT or vice versa) did not significantly influence the responses to the low-volume CT intervention. (2) As hypothesized, when pooling data by modality, LOW-HIIT combined with 1-RT showed superior improvements in cardiometabolic health, inflammation, and some physical fitness measures compared to LOW-HIIT combined with WB-EMS.

To date, only a very limited number of studies have addressed CT order effects on cardiometabolic outcomes in clinical cohorts. Aligning with our results, Delgado-Floody et al. [52] observed similar benefits for glucose, triglycerides, and blood pressure in morbidly obese women, regardless of training order, after 20 weeks of HIIT combined with multiple-set RT. These findings suggest that in obese populations, initial improvements in cardiometabolic outcomes can occur regardless of the specific sequence of exercises, as long as a consistent training stimulus is applied.

Although we did not observe significant order effects in either CT combination, subtle differences warrant brief discussion. In the LOW-HIIT and WB-EMS combination, reductions in WC and FM were significant only when WB-EMS preceded LOW-HIIT, possibly indicating an enhanced lipolytic response with this exercise order. Conversely, a significant reduction in hsCRP was only evident in the LOW-HIIT+WB-EMS group, suggesting that initiating the session with cardiovascular activity prior to WB-EMS may elicit greater anti-inflammatory adaptations. In the LOW-HIIT and 1-RT combination, significant reductions in FM%, HbA_1c_, triglycerides, insulin, and HOMA-IR were observed only when 1-RT was performed first, indicating a potential benefit for glycemic control and lipid metabolism when starting the session with RT. However, as no significant between-group differences were found in both exercise combinations, these interpretations remain speculative, and further studies with larger samples are needed to verify these trends.

With respect to anthropometric changes, some findings require discussion. First, it became evident that the mean weight loss of ~2.1 kg across all intervention groups occurred under a reported caloric deficit of 323–483 kcal/day, which was slightly below the planned 500 kcal/day. This aligns with well-known challenges in maintaining long-term adherence to calorie restriction, as seen in prior weight loss interventions [53,54]. Second, the expected average weight loss of approximately 0.3–0.5 kg/week (based on the self-reported caloric deficit) was not achieved, likely due to underreporting of dietary intake. Underreporting is a well-documented issue in dietary self-monitoring, with underestimation of caloric intake ranging from 10% to 45% [55,56]. It was reported that underreporting increases with higher BMI, most likely due to intentional or unintentional omission of certain food items or portion sizes [55]. Other errors in food records can stem from intentional underreporting influenced by social desirability or difficulties recording foods eaten outside the home [57]. Additionally, adaptive thermogenesis—a reduction in energy expenditure in response to repeated weight loss attempts—may diminish the expected weight loss despite dietary adherence [58]. Third, the modest weight loss observed in all groups suggests that low-volume exercise modalities may have a limited impact on overall energy expenditure compared to higher-volume regimens [59]. Accordingly, Keating et al. [60] reported that lower-volume HIIT protocols result in a smaller energy deficit compared to higher-volume ET. Likewise, a typical 20 min WB-EMS session has been found to expend less energy than traditional multiple-set RT [61].

However, despite only modest weight loss, significant reductions in FM% and WC occurred in both pooled groups. The observed reductions in WC (−1.3 cm to −3.9 cm) have clinical relevance. Epidemiological data have shown that each 1 cm reduction in WC correlates with a 2–3% decrease in cardiovascular disease and all-cause mortality risk [62], with benefits persisting independently of BMI [63]. Moreover, the low-volume CT programs preserved FFM, a key factor for weight maintenance and metabolic health. This finding aligns with evidence that both resistance exercise and HIIT stimulate muscle protein synthesis and thereby mitigate the catabolic effects of caloric restriction [64,65].

As expected, both low-volume CT combinations improved cardiorespiratory and muscular fitness, with increases in VO_2max_, W_VT_, and 1-RM across all major muscle groups. These improvements are clinically important, as they are linked to reduced morbidity and mortality [66,67]. For example, even a modest increase in VO_2max_ by 1.0 mL/kg/min has been associated with a ~9% reduction in cardiovascular disease mortality risk [68]. Thus, the observed VO_2max_ increases in this study (+1.4 to +2.1 mL/kg/min) can be deemed clinically meaningful, especially for individuals with low initial cardiorespiratory fitness levels. Improvements in W_VT_, a key marker of submaximal aerobic capacity, enhance metabolic efficiency, including lactate clearance and muscle fatigue delay, and are associated with better exercise tolerance and lower risk of cardiovascular events [69,70]. The observed 1-RM increases in all major muscle groups of up to 35 kg are notable and likely to translate into improved overall health and quality of life. Even small 1-RM gains of 1–2 kg have been reported to improve daily function, reduce fall risk, and enhance physical autonomy [71,72]. Additionally, strength improvements have been linked to several psychological benefits, such as greater self-efficacy, reduced anxiety, and better mental well-being [71]. Taken together, the significant Fit-score improvements highlight that low-volume CT programs can effectively enhance overall fitness with a relatively small time investment (~65 min/week), which is crucial given that time commitment strongly influences adherence in previously untrained individuals [73]. In this respect, it should be noted that both low-volume CT combinations improved cardiorespiratory and muscular fitness parameters, but the pooled LOW-HIIT and 1-RT group showed greater overall fitness gains, mainly due to greater increases in abdominal and lower back muscle strength. This aligns with prior research [22,74], suggesting that traditional RT leads to superior strength adaptations compared to WB-EMS, at least in certain muscle groups.

Consistent with previous research on low-volume exercise [19,20,21,22,23,26], the pooled LOW-HIIT and 1-RT group showed significant improvements in cardiometabolic and inflammation markers, likely associated with clinical benefits. The observed blood pressure reduction by an average of −8 mmHg systolic and −6 mmHg diastolic is comparable to pharmacological interventions [75]. This finding is meaningful, as every 5 mmHg decrease has been related to a 10% lower risk of cardiovascular events [76]. The significant decrease in resting heart rate, a prognostic marker for cardiovascular disease risk [77], suggests improved cardiovascular efficiency following the intervention. In line with our previous research [20,23], we also observed a marked reduction in inflammation markers, with hsCRP levels dropping by ~37% after 12 weeks of LOW-HIIT and 1-RT. This finding is comparable to reductions seen in a pharmacological trial applying an interleukin-1β blocker for 48 months [78]. Given the strong link between chronic low-grade inflammation and major health risks [6,79], these findings are promising and highlight the potential of low-volume exercise as a viable non-pharmacological strategy for improving inflammation status. The reduction in inflammation was accompanied by significant improvements in key biochemical risk markers, including HbA_1c_, triglycerides, and HDL, which may independently lower cardiovascular risk [80,81,82]. Furthermore, the substantial drop in the MetS z-score indicates a meaningful reduction in MetS severity, highlighting significant improvements in overall cardiometabolic health following LOW-HIIT and 1-RT.

It was a key finding that the cardiometabolic and anti-inflammatory benefits seen in the pooled LOW-HIIT and 1-RT group were largely absent in the LOW-HIIT and WB-EMS group. While the physiological mechanisms remain speculative, several factors may explain these differences. It is well established that traditional RT can improve vascular function, reduce arterial stiffness, and lower blood pressure by enhancing nitric oxide bioavailability and vascular compliance [83]. RT has also been shown to reduce inflammation through anti-inflammatory myokine release and improve other cardiometabolic risk factors [84,85]. In contrast, the effects of WB-EMS on these outcomes are less clear [86]. These variations may stem from differences in muscle fiber activation and subsequent physiological adaptations between conventional RT and WB-EMS [22]. Further, when compared to previous findings from our laboratory, where LOW-HIIT, WB-EMS, or 1-RT were performed alone under identical conditions in a MetS collective [20] (Figure 4), the combination of LOW-HIIT and 1-RT appears to further enhance reductions in inflammation. This may result from their complementary mechanisms: HIIT primarily lowers pro-inflammatory markers, while RT promotes anti-inflammatory cytokine secretion [84,87]. These synergistic effects may likely amplify the anti-inflammatory impact, explaining the superior response seen with their combination. In contrast, the LOW-HIIT and WB-EMS combination showed a weaker impact on cardiometabolic health, as indicated by a smaller reduction in the MetS z-score compared to single low-volume exercises [20]. This suggests a potential interference effect on cardiometabolic adaptations when combining LOW-HIIT with WB-EMS. Possible factors include antagonistic molecular responses [27,28], divergent pathway activation impairing muscle fiber adaptations [27], and conflicting neuromuscular demands [28]. Further research is needed to confirm this potential interference and identify the underlying physiological mechanisms.

## 5. Strengths and Limitations

This study has several notable strengths. It is the first trial to compare different low-volume CT regimens on fitness, cardiometabolic, and inflammatory markers in obese MetS patients, addressing the need for feasible and effective exercise programs for chronic disease populations. The low-volume CT programs used in this study are simple, replicable, and well-tolerated by individuals at increased cardiometabolic risk, as indicated by high adherence rates, low dropout rates, and an excellent safety profile. Robust assessment methods, including CPET for VO_2max_, standardized strength tests, and validated laboratory analyses, ensured objective data collection by blinded evaluators. Additionally, standardized nutritional counseling minimized dietary confounding, strengthening the validity of between-group comparisons.

However, there are also some limitations to consider. First, the interventions were conducted in a controlled clinical setting under close supervision, which may not fully reflect real-world conditions. While the high adherence suggests feasibility in other settings, further research is needed to confirm applicability in rehabilitation clinics, fitness centers, and home-based programs. Second, the absence of a passive control group limits the ability to quantify the specific benefits of low-volume CT compared to no exercise. However, extensive evidence consistently supports the superiority of exercise over passive control groups for improving fitness, cardiometabolic health, and inflammation in obese and MetS cohorts [20,21,22,23,26,88,89]. Third, the study lasted 12 weeks, so the long-term effects of the interventions remain uncertain. Future research should explore the sustainability and long-term impact of combining LOW-HIIT with 1-RT or WB-EMS on health outcomes. Fourth, we recognize that caloric restriction, as part of the study protocol, could introduce bias. All patients received standard nutritional counseling for moderate caloric restriction, which may have contributed to the observed improvements in several health outcomes to some degree. However, caloric restriction is a well-established part of standard therapy for obesity and MetS, widely recommended in clinical guidelines. Omitting it would have been neither appropriate nor ethical, as it is a known intervention for improving health outcomes in MetS populations.

## 6. Conclusions

Our results showed that the order of execution—whether LOW-HIIT preceded WB-EMS or 1-RT, or vice versa—had no significant effect on anthropometric, cardiometabolic, or physical fitness outcomes in obese individuals with MetS. When comparing the pooled CT groups, the LOW-HIIT and 1-RT combination showed greater benefits for cardiometabolic health, inflammation, and overall physical fitness than the LOW-HIIT and WB-EMS combination. Therefore, combining LOW-HIIT with 1-RT appears to be the superior exercise option compared to the LOW-HIIT and WB-EMS combination and should be prioritized when both options are available. However, LOW-HIIT combined with WB-EMS still offers meaningful health and fitness improvements and can be a viable alternative for individuals unable to perform traditional RT, such as those with mobility limitations. Further research is needed to investigate potential modality-related interference effects on cardiometabolic health between LOW-HIIT and WB-EMS.

## Figures and Tables

**Table 1:** Main baseline characteristics of the four study groups (including dropouts).

Outcome	LOW-HIIT+ WB-EMS (*n* = 30)	WB-EMS+ LOW-HIIT (*n* = 31)	LOW-HIIT+ 1-RT (*n* = 30)	1-RT+ LOW-HIIT (*n* = 30)
Age (years)	50.8 ± 11.5	48.4 ± 13.6	50.8 ± 10.0	50.1 ± 11.0
Gender, male/female (*n*)	12/18	11/20	14/16	12/18
BMI (kg/m^2^)	37.8 ± 5.6	39.3 ± 5.4	38.4 ± 7.8	38.2 ± 6.5
MetS z-score	2.10 ± 2.35	2.43 ± 3.26	2.47 ± 3.47	2.36 ± 2.51
VO_2max_ (mL/kg/min)	20.7 ± 5.5	19.9 ± 5.2	21.9 ± 6.8	21.7 ± 4.7
Fit-score	40 ± 12	39 ± 12	41 ± 11	40 ± 10

Data are shown as mean ± SD. BMI = body mass index; MetS z-score = metabolic syndrome severity z-score; VO_2max_ = maximum oxygen uptake; Fit-score = overall fitness score.

**Table 2:** Dietary intakes during week 0 and week 12.

Outcome	LOW-HIIT+ WB-EMS (*n* = 23)		WB-EMS+ LOW-HIIT (*n* = 22)		LOW-HIIT+ 1-RT (*n* = 25)		1-RT+ LOW-HIIT (*n* = 23)	
	Week 0	Week 12	Week 0	Week 12	Week 0	Week 12	Week 0	Week 12
Nutrition								
Energy (kcal/d)	2224 ± 467	1901 ± 438 ^c^	2389 ± 666	1966 ± 703 ^c^	2568 ± 676	2174 ± 632 ^c^	2204 ± 690	1720 ± 559 ^c^
Protein (g/kg/d)	0.9 ± 0.3	0.9 ± 0.2	0.9 ± 0.3	0.8 ± 0.3	1.1 ± 0.4	1.0 ± 0.5	0.9 ± 0.3	0.8 ± 0.3
Fat (g/kg/d)	0.9 ± 0.3	0.7 ± 0.2 ^b^	0.9 ± 0.4	0.7 ± 0.4 ^a^	0.9 ± 0.3	0.8 ± 0.2 ^b^	0.8 ± 0.3	0.7 ± 0.3 ^c^
CHO (g/kg/d)	2.1 ± 0.5	1.7 ± 0.7 ^b^	2.0 ± 0.7	1.7 ± 0.7 ^a^	2.5 ± 1.0	2.0 ± 0.7 ^c^	2.0 ± 0.6	1.7 ± 0.6 ^b^
Fibres (g/d)	26 ± 14	26 ± 13	21 ± 9	20 ± 7	25 ± 7	23 ± 7 ^c^	22 ± 9	21 ± 8

Data are shown as mean ± SD. Week 0 = 1 week before the study began; Week 12 = final intervention week; CHO = carbohydrates. ^a^ (*p* < 0.05), ^b^ (*p* < 0.01), ^c^ (*p* < 0.001): significant difference vs. Week 0.

**Table 3:** Study outcomes pre- and post-intervention in the LOW-HIIT and WB-EMS groups.

	LOW-HIIT+ WB-EMS (*n* = 23)		WB-EMS+ LOW-HIIT (*n* = 22)	
Variable	T-1	T-2	T-1	T-2
Age (years)	51.3 ± 12.3	---	49.3 ± 14.9	---
Gender, male/female (*n*)	11/12	---	7/15	---
Anthropometric variables				
Body weight (kg)	108.6 ± 17.9	107.0 ± 16.6 ^a^	115.1 ± 23.4	113.7 ± 23.4 ^a^
Body mass index (kg/m^2^)	38.1 ± 5.7	37.6 ± 5.3 ^a^	40.4 ± 5.9	39.9 ± 5.9 ^a^
Fat mass (kg)	47.5 ± 12.3	46.8 ± 11.3	54.4 ± 13.3	53.3 ± 12.7 ^a^
Body fat (%)	43.5 ± 7.2	43.1 ± 7.0	47.0 ± 5.1	46.6 ± 5.5
Fat-free mass (kg)	61.1 ± 11.0	60.4 ± 11.3	60.7 ± 12.7	60.3 ± 13.6
Total body water (L)	45.5 ± 7.7	45.1 ± 8.0	45.5 ± 9.2	45.1 ± 10.0
Waist circumference (cm)	114 ± 12	113 ± 11	116 ± 14	114 ± 14 ^a^
Blood pressure				
Systolic blood pressure (mmHg)	134 ± 15	129 ± 13	133 ± 14	133 ± 13
Diastolic blood pressure (mmHg)	87 ± 8	85 ± 7	86 ± 8	85 ± 9
MAB (mmHg)	103 ± 8	99 ± 8	101 ± 12	100 ± 8
Resting heart rate (b/min)	75 ± 10	71 ± 8 ^a^	75 ± 10	74 ± 10
Clinical chemistry				
CRP (mg/L)	5.0 ± 3.3	3.8 ± 2.0	5.4 ± 4.1	5.1 ± 3.7
hsCRP (mg/L)	3.8 ± 2.6	2.8 ± 1.6 ^b^	4.2 ± 3.2	3.9 ± 3.0
Glucose (mg/dL)	106 ± 14	108 ± 14 ^a^	109 ± 21	111 ± 23
HbA_1c_ (%)	5.7 ± 0.5	5.7 ± 0.5	5.7 ± 0.6	5.7 ± 0.6
Insulin (µE/mL)	18 ± 8	17 ± 9	20 ± 11	22 ± 12
HOMA-IR (units)	4.6 ± 2.3	4.6 ± 2.6	5.5 ± 3.7	5.7 ± 4.1
Triglycerides (mg/dL)	139 ± 48	160 ± 83	135 ± 77	126 ± 66
Cholesterol (mg/dL)	216 ± 35	213 ± 34	225 ± 42	223 ± 43
HDL (mg/dL)	51 ± 10	50 ± 10	55 ± 13	54 ± 13
LDL (mg/dL)	144 ± 28	140 ± 26	147 ± 32	148 ± 33
MetS z-score (units)	2.3 ± 2.2	2.7 ± 2.3	2.6 ± 3.7	2.7 ± 3.7
CPET variables				
VO_2max_ (L)	2.23 ± 0.57	2.39 ± 0.58 ^b^	2.17 ± 0.61	2.28 ± 0.64 ^b^
VO_2max_ (mL/kg/min)	21.0 ± 5.9	22.6 ± 5.5 ^b^	19.1 ± 5.0	20.3 ± 5.2 ^b^
W_max_ (W)	159 ± 39	181 ± 41 ^c^	158 ± 41	179 ± 48 ^c^
W_VT_ (W)	72 ± 11	87 ± 16 ^c^	70 ± 18	85 ± 25 ^c^
Muscle strength				
1-RM abdominals (kg)	28 ± 9	33 ± 9 ^c^	27 ± 10	32 ± 11 ^c^
1-RM lower back (kg)	58 ± 26	67 ± 30 ^a^	57 ± 21	71 ± 32 ^c^
1-RM chest (kg)	34 ± 12	43 ± 16 ^c^	36 ± 21	44 ± 20 ^c^
1-RM upper back (kg)	48 ± 10	58 ± 12 ^c^	50 ± 18	59 ± 21 ^c^
1-RM legs (kg)	147 ± 64	174 ± 82 ^c^	128 ± 45	150 ± 63 ^c^
Fit-score (units)	42 ± 12	49 ± 14 ^c^	39 ± 12	46 ± 16 ^c^

Data are shown as mean ± SD. MAB = mean arterial blood pressure; CRP = *C*-reactive protein; hsCRP = high-sensitivity *C*-reactive protein; HbA_1c_ = glycosylated haemoglobin A_1c_; HOMA-IR = homeostasis model assessment of insulin resistance; HDL/LDL = high-/low-density lipoprotein cholesterol; MetS z-score = metabolic syndrome severity score; CPET = cardiopulmonary exercise testing; VO_2max_ = maximal oxygen uptake; W_max_ = maximal power output; W_VT_ = power output at the ventilatory threshold; 1-RM = one-repetition maximum strength; Fit-score = overall fitness score. ^a^ (*p* < 0.05), ^b^ (*p* < 0.01), ^c^ (*p* < 0.001): significant difference vs. T-1.

**Table 4:** Study outcomes pre- and post-intervention in the LOW-HIIT and 1-RT groups.

	LOW-HIIT+ 1-RT (*n* = 25)		1-RT+ LOW-HIIT (*n* = 23)	
Variable	T-1	T-2	T-1	T-2
Age (years)	51.8 ± 10.0	---	51.5 ± 11.1	---
Gender, male/female (*n*)	11/14	---	10/13	---
Anthropometric variables				
Body weight (kg)	112.1 ± 25.8	110.1 ± 26.6 ^c^	107.4 ± 19.2	103.6 ± 19.1 ^b^
Body mass index (kg/m^2^)	37.8 ± 7.9	37.1 ± 8.1 ^c^	36.2 ± 4.4	35.0 ± 4.9 ^b^
Fat mass (kg)	48.7 ± 16.9	47.5 ± 17.7 ^a^	45.1 ± 8.6	42.0 ± 10.1 ^a^
Body fat (%)	42.9 ± 9.6	42.4 ± 9.7	42.2 ± 5.0	40.6 ± 6.6 ^a^
Fat-free mass (kg)	63.4 ± 15.5	62.6 ± 15.1	62.3 ± 13.4	61.6 ± 13.3
Total body water (L)	47.2 ± 11.0	46.7 ± 11.0	46.6 ± 9.6	45.9 ± 9.5
Waist circumference (cm)	112 ± 17	109 ± 18 ^a^	111 ± 13	106 ± 12 ^c^
Blood pressure				
Systolic blood pressure (mmHg)	136 ± 12	129 ± 10 ^c^	134 ± 13	123 ± 10 ^c^
Diastolic blood pressure (mmHg)	89 ± 7	83 ± 7 ^c^	90 ±9	82 ± 6 ^c^
MAB (mmHg)	105 ± 6	99 ± 7 ^c^	104 ± 10	96 ± 6 ^c^
Resting heart rate (b/min)	74 ± 12	71 ± 10 ^a^	78 ± 10	73 ± 11 ^a^
Clinical chemistry				
CRP (mg/L)	6.8 ± 5.5	4.8 ± 4.2 ^c^	6.0 ± 7.5	2.5 ± 1.9 ^a^
hsCRP (mg/L)	5.6 ± 5.0	3.6 ± 3.4 ^c^	4.9 ± 6.8	1.8 ± 1.5 ^a^
Glucose (mg/dL)	104 ± 20	104 ± 15	103 ± 13	102 ± 10
HbA_1c_ (%)	5.6 ± 0.5	5.5 ± 0.4	5.4 ± 0.8	5.2 ± 0.7 ^a^
Insulin (µE/mL)	16 ± 10	15 ± 14	19 ± 14	14 ± 5 ^b^
HOMA-IR (units)	4.3 ± 3.4	4.0 ± 4.5	5.1 ± 4.6	3.5 ± 1.5 ^a^
Triglycerides (mg/dL)	145 ± 56	134 ± 49	132 ± 67	109 ± 46 ^a^
Cholesterol (mg/dL)	232 ± 46	229 ± 41	225 ± 42	223 ± 43
HDL (mg/dL)	52 ± 7	54 ± 6	53 ± 13	56 ± 16 ^a^
LDL (mg/dL)	155 ± 36	155 ± 35	143 ± 35	137 ± 33
MetS z-score (units)	2.4 ± 3.5	1.0 ± 3.4 ^c^	1.9 ± 2.4	0.1 ± 1.8 ^c^
CPET variables				
VO_2max_ (L)	2.38 ± 0.65	2.54 ± 0.61 ^b^	2.37 ± 0.48	2.57 ± 0.56 ^b^
VO_2max_ (mL/kg/min)	22.0 ± 7.2	23.9 ± 6.8 ^c^	22.8 ± 4.4	25.2 ± 4.2 ^c^
W_max_ (W)	170 ± 44	202 ± 48 ^c^	179 ± 36	207 ± 46 ^c^
W_VT_ (W)	77 ± 22	102 ± 27 ^c^	73 ± 9	95 ± 21 ^c^
Muscle strength				
1-RM abdominals (kg)	32 ± 13	41 ± 14 ^c^	32 ± 11	42 ± 13 ^c^
1-RM lower back (kg)	59 ± 27	80 ± 33 ^c^	53 ± 17	73 ± 27 ^c^
1-RM chest (kg)	36 ± 16	48 ± 21 ^c^	36 ± 16	43 ± 17 ^c^
1-RM upper back (kg)	49 ± 19	62 ± 21 ^c^	50 ± 18	62 ± 18 ^c^
1-RM legs (kg)	125 ± 38	167 ± 63 ^c^	131 ± 40	159 ± 50 ^c^
Fit-score (units)	41 ± 11	53 ± 15 ^c^	41 ± 9	50 ± 11 ^c^

Data are shown as mean ± SD. MAB = mean arterial blood pressure; CRP = *C*-reactive protein; hsCRP = high-sensitivity *C*-reactive protein; HbA_1c_ = glycosylated haemoglobin A_1c_; HOMA-IR = homeostasis model assessment of insulin resistance; HDL/LDL = high-/low-density lipoprotein cholesterol; MetS z-score = metabolic syndrome severity score; CPET = cardiopulmonary exercise testing; VO_2max_ = maximal oxygen uptake; W_max_ = maximal power output; W_VT_ = power output at the ventilatory threshold; 1-RM = one-repetition maximum strength; Fit-score = overall fitness score. ^a^ (*p* < 0.05), ^b^ (*p* < 0.01), ^c^ (*p* < 0.001): significant difference vs. T-1.

## Data Availability

The datasets generated and analyzed during the current study are not publicly available but are available from the corresponding author upon reasonable request.

## Supplementary Materials

The following supporting information can be downloaded at: https://www.mdpi.com/article/10.3390/nu17030561/s1. Table S1: Concomitant diseases and medication in the four study groups.
