# Supplementary material for: Impact of Different Low-Volume Concurrent Training Regimens on Cardiometabolic Health, Inflammation, and Fitness in Obese Metabolic Syndrome Patients
Source: Nutrients. 2025 Jan 31;17(3):561. doi: 10.3390/nu17030561 (PMC11820124; doi:10.3390/nu17030561)
Supplement: Supplementary file 1 [file nutrients-17-00561-s001.zip › nutrients-3418939-supplementary.pdf]

Supplementary Table S1: Concomitant diseases and medication in the four study groups

	**LOW-HIIT+ WB-EMS (*n* = 23)**	**WB-EMS+ LOW-HIIT (*n* = 22)**	**LOW-HIIT+ 1-RT (*n* = 25)**	**1-RT+ LOW-HIIT (*n* = 23)**
Condition, *n* (%)				
Hypertension	20 (87%)	20 (91%)	23 (92%)	20 (87%)
Dyslipidemia	21 (91%)	19 (86%)	23 (92%)	20 (87%)
Hyperglycemia / pre-diabetes	11 (48%)	14 (64%)	12 (48%)	19 (48%)
Low grade inflammation	23 (100%)	21 (96%)	24 (96%)	22 (96%)
Asthma	2 (9%)	0 (0%)	3 (12%)	3 (13%)
Gastrointestinal disorder	7 (30%)	5 (23%)	5 (20%)	5 (22%)
Urogenital disorder	0 (0%)	0 (0%)	1 (4%)	3 (13%)
Allergic disorder	4 (17%)	5 (23%)	2 (8%)	7 (30%)
Thyroid disorder	3 (13%)	5 (23%)	5 (20%)	4 (17%)
Hepatic disorder	2 (9%)	3 (14%)	4 (16%)	2 (9%)
Hyperuricemia / gout	9 (39%)	9 (41%)	12 (48%)	7 (30%)
Migraine	3 (13%)	5 (23%)	4 (16%)	5 (22%)
Tinnitus	5 (22%)	4 (18%)	5 (20%)	5 (22%)
Sleep apnea	1 (4%)	1 (5%)	0 (0%)	1 (4%)
Musculoskeletal / joint disorder	14 (61%)	14 (64%)	15 (60%)	15 (65%)
Neurological disorder	0 (0%)	0 (0%)	1 (4%)	0 (0%)
Mental disorder / depression	7 (30%)	10 (45%)	11 (44%)	6 (26%)
Medication, *n* (%)				
Antihypertensives	15 (65%)	12 (55%)	12 (48%)	12 (52%)
Statins	3 (13%)	3 (14%)	3 (12%)	2 (9%)
Metformin	4 (17%)	3 (14%)	2 (8%)	2 (9%)
Analgesics	14 (61%)	11 (50%)	10 (40%)	11 (48%)
L-thyroxine	1 (4%)	3 (14%)	2 (8%)	1 (4%)
Antihistamines	1 (4%)	2 (9%)	1 (4%)	4 (17%)
Bronchodilators	2 (9%)	0 (0%)	2 (8%)	1 (4%)
Sedatives	2 (9%)	3 (14%)	2 (8%)	1 (4%)
Anti-depressants	2 (9%)	2 (9%)	0 (0%)	4 (17%)
